# Programas de garantía externa de la calidad SEQC^ML^. Evolución de las prestaciones analíticas de los laboratorios clínicos a lo largo de 30 años y comparación con otros programas

**DOI:** 10.1515/almed-2019-0024

**Published:** 2020-05-04

**Authors:** Carmen Perich Alsina, Carmen Ricós, Fernando Marqués, Joana Minchinela, Angel Salas, Cecilia Martínez-Bru, Beatriz Boned, Rubén Gómez-Rioja, Marià Cortés, Elisabet González-Lao, Jose Vicente García Lario, Xavier Tejedor, Sandra Bullich, Montserrat Ventura, Ricardo González-Tarancón, Pilar Fernández-Fernández, Francisco Ramón, Zoraida Corte, Maria Antonia Llopis, Jorge Díaz-Garzón, Margarita Simón, Pilar Fernández-Calle

**Affiliations:** Comité de Programas Externos de la SEQC^ML^ , Barcelona, España; Comisión de Calidad Analítica de la SEQC^ML^ , Barcelona, España; Comité de Programas Externos de la SEQC^ML^ , C/Aribau 263, 08021, Barcelona, España

**Keywords:** armonización, especificaciones de prestación analítica, estado del arte, programas de garantía externa de la calidad, variación biológica

## Abstract

**Objetivos:**

El objetivo de este estudio es conocer la evolución de la prestación analítica de los laboratorios participantes en los programas EQA de la SEQC^ML^ durante los 30 años de funcionamiento y compararla con la prestación obtenida en otros programas EQA para saber si los resultados son similares.

**Métodos:**

Se evalúan los resultados obtenidos durante este periodo, aplicando las especificaciones de la calidad derivadas de la VB y del estado del arte. Además, se realiza una comparación con los resultados obtenidos por otras organizaciones de programas EQA.

**Resultados:**

Se observa que los laboratorios participantes en los programas EQA-SEQC^ML^ han mejorado su prestación durante los 30 años de experiencia y que las especificaciones derivadas de la variación biológica son alcanzables. La comparación entre programas EQA es difícil, debido a: la falta de accesibilidad y a las diferencias en el diseño de estos programas (materiales control, cálculos empleados y especificaciones analíticas establecidas).

**Conclusiones:**

Los datos de este estudio ponen de manifiesto que para algunas magnitudes biológicas los resultados obtenidos en los programas todavía no están armonizados, aunque se estan realizando esfuerzos para alcanzar la armonización. Los organizadores de programas EQA deberían sumarse al esfuerzo de armonización, facilitando la información sobre sus resultados para permitir su comparación.

## Introducción

Los programas de garantía externa de la calidad (EQA) son un componente fundamental de la gestión de la calidad de los laboratorios. Su participación permite al laboratorio evaluar y monitorizar su prestación analítica, comparar la fiabilidad de los procedimientos de medida y conocer el grado de armonización de los resultados analíticos.

La Sociedad Española de Medicina de Laboratorio (SEQC^ML^) puso en marcha su primer programa EQA en1981, incluyendo 20 magnitudes bioquímicas en suero y con 147 participantes [1]. Desde entonces, se han incorporado programas propios y se han establecido colaboraciones con programas nacionales y europeos (Sociedad Española de Hematología y Hemoterapia para magnitudes hematológicas, UK NEQAS para las de inmunología y alergia, y OELM para elementos traza). En la actualidad, la SEQC^ML^ distribuye 29 programas que abarcan todas las áreas del laboratorio, cubriendo 189 magnitudes biológicas y en los que participan alrededor de 700 laboratorios.

La información que ha de facilitar un programa EQA según el *Task Group on performance specifications for EQAS* de la European Federation of Clinical Chemistry and Laboratory Medicine (EFLM-TG-EQA) creado en la 1^a^ Conferencia estratégica de Milán en 2014 [2], para que los participantes puedan interpretar correctamente sus resultados [3] concierne a: el material control distribuido, el valor diana al que se compara el resultado del laboratorio, cómo se expresa la prestación analítica del participante y el criterio de evaluación utilizado.

El material control de la mayoría de los programas EQA de la SEQC^ML^ es suero estabilizado excepto el programa de suero conmutable con valores asignados por método de referencia (SCR) que utiliza suero humano congelado.

El valor diana de las muestras control es el valor medio obtenido por cada grupo de usuarios del mismo método analítico e instrumento (grupo homogéneo o grupo par), y el del programa SCR es el valor de referencia certificado.

La prestación analítica del laboratorio se expresa mediante la desviación porcentual (DP%) de cada resultado respecto al valor diana, indicativo del error total (ET) analítico. La prestación analítica de los métodos participantes se expresa mediante el coeficiente de variación (CV) inter-laboratorios , que indica la imprecisión de cada grupo homogéneo y mediante la DP% de la media de cada grupo homogéneo que indica el sesgo del método.

El criterio de evaluación ha variado con los años: hasta 1993 se usaba el criterio estadístico de la puntuación estándar respecto al grupo homogéneo [4]. A partir de 1994 se aplicó, además, la especificación de calidad analítica (EPA) derivada de la variabilidad biológica (VB) cuando existían datos disponibles [5] y en caso contrario, a partir del 2011, el percentil 90 de los resultados de las desviaciones individuales (DP%) obtenidas por los participantes, una vez excluidos los valores extremos.

En el caso de utilizar VB, se emplean tres “niveles” de exigencia, denominados mínimo, deseable y óptimo [6], según el porcentaje de desviaciones individuales de los laboratorios que cumplen la especificación:

Mínimo: menos del 80% de las DP lo cumplirían
Deseable : entre un 80 y un 90%
Óptimo: un 90% o más DP estarían dentro del límite definido por la VB.

Siguiendo la tipificación de los programas EQA descrita por Miller y cols [7] la mayor parte de los programas de la SEQC^ML^ son de categoría 4 (material control de suero humano estabilizado, valor asignado por el grupo homogéneo, medidas repetidas, EPA basadas en la VB); el programa SCR es de categoría 1 (material control de suero humano congelado, valor asignado por método de referencia, medidas repetidas, EPA basadas en la VB).

Los objetivos de este estudio son:

conocer la evolución de la prestación analítica de los laboratorios participantes en los programas EQA de la SEQC^ML^ a lo largo de más de 30 años de funcionamiento (1981 – 2018), y
comparar la prestación analítica obtenida por los laboratorios participantes en los programas EQA de la SEQC^ML^ con la de los participantes en otros programas EQA.

## Materiales y métodos

El material empleado en este estudio son los informes para el laboratorio individual y de final de ciclo, que los programas EQA de la SEQC^ML^ proporcionan a los participantes.

### Informe para el laboratorio individual

Ha evolucionado con el tiempo, cumpliendo actualmente las recomendaciones del EFLM-TF-EQA [3]. Los datos incluidos son:

histograma de distribución de resultados
número de resultados recibidos
número de resultados incluidos en los cálculos estadísticos (se excluyen los valores aberrantes, que exceden el intervalo media ± 3 desviaciones estándar reiterativamente hasta que no queda ningún dato fuera de dicho intervalo) [8]
media y desviación estándar de todos los participantes, del mismo método que el participante y del mismo método e instrumento (grupo homogéneo),
resultado obtenido por el laboratorio y su desviación respecto al valor diana, expresada en puntuación estándar y en porcentaje
desviación porcentual aceptable según el criterio derivado de la VB para el ET analítico, habiéndose añadido en el año 2005 una gráfica con los resultados de los últimos 12 meses y su desviación respecto a la VB (Supplementary Figura 1).

En el programa SCR el informe incluye:

desviación porcentual respecto al valor de referencia de las six muestras,
coeficiente de variación (obtenido entre los replicados control) del laboratorio,
media, coeficiente de variación y desviación porcentual de la media del grupo homogéneo (mismo método, instrumento y trazabilidad) respecto al valor de referencia (Supplementary Figura 2).

Siguiendo su objetivo educativo, la organización elabora y envía notas técnicas relacionadas con los programas para ayudar a los participantes en la interpretación de los resultados. Asimismo, mantiene una comunicación bidireccional para cualquier consulta que los laboratorios participantes deseen realizar.

### Informe de fin de ciclo

Contiene los indicadores de la prestación analítica de los métodos participantes:

Coeficiente de variación inter-laboratorios de los grupos homogéneos.
Sesgo de los grupos homogéneos (diferencia entre la media del grupo y el valor diana).
Percentiles de la desviación de cada resultado respecto al grupo homogéneo (DP%), desde el año 2011. Inicialmente se mostró el percentil 90 (P90) pero, a partir del año 2017, se muestran también P20, P50 y P70 para que cada laboratorio pueda seleccionar su especificación.

El método utilizado es la inspección visual de los informes de fin de ciclo emitidos desde 1981 hasta 2018, la cuantificación del porcentaje de resultados que cumplen las EPA y la comparación visual de los indicadores con respecto a los de programas de otros países.

## Resultados

### Evolución de la prestación analítica de los laboratorios participantes en los programas EQA de la SEQC^ML^

**Figura 1:**
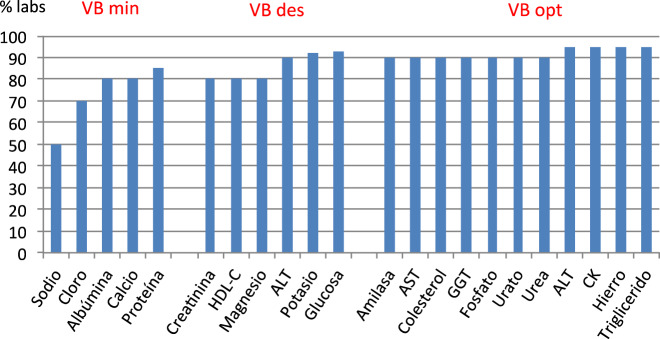
Cumplimiento de la especificación para error total analítico derivada de la VB en el programa de Bioquímica suero del 2018.

A partir del informe individual se obtiene la desviación respecto al grupo par. En la Figura 1 se muestra el porcentaje de resultados individuales de los laboratorios (obtenida del informe individual) en el año 2018, cuya desviación (DP%) respecto al grupo homogéneo cumple con la especificación de calidad para ET analítico derivada de la VB (mínima, deseable u óptima), en el programa de bioquímica en suero.

Para la mayoría de las magnitudes biológicas, el 90% de los resultados de los participantes cumplen con la especificación e incluso para las magnitudes con una mayor regulación homeostática (menor VB y especificaciones más restrictivas), el 50% o más de los laboratorios es ya capaz de cumplir el requisito.

Si se compara la DP% del percentil 90 de los laboratorios participantes en el programa de hormonas entre los años 2012 y 2018, se observa una disminución muy importante (Supplementary Figura 3). El 90% de los resultados de insulina de los laboratorios en el año 2012 obtenía DP = 39% que ha disminuido a DP = 14% en el 2018. Asimismo, el P90 de los resultados de péptido C ha disminuido de 23% a 14% y los de tirotropina del 13% al 9%.

**Figura 2:**
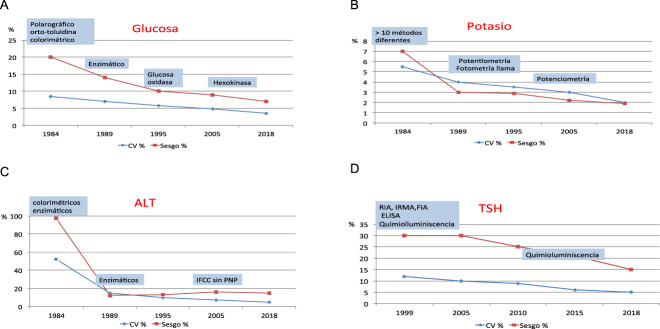
Evolución de la imprecisión inter-laboratorios y del sesgo respecto al grupo homogéneo.

A partir del informe de fin de ciclo se obtienen los indicadores de imprecisión inter-laboratorios (medida como CV) y sesgo respecto al grupo homogéneo (medido como DP%). En la Figura 2 se muestran cinco cortes temporales de magnitudes escogidas como representativas de sustratos (glucosa), electrolitos (potasio), enzimas (ALT) y hormonas (TSH)

Glucosa (Figura 2A): el uso mayoritario de los métodos enzimáticos a partir de los años 90 disminuyó la imprecisión inter-laboratorios al 6% y el sesgo al 10%. Con el uso de métodos enzimáticos mas específicos, en el año 2018 se obtuvo un CV del 4% y una DP del 7%.
Potasio (Figura 2B): en el año 1984 existían más de diez métodos diferentes para analizar potasio, con un CV del 5,5% y un sesgo del 7%; desde 1989 hasta el año 2000 coexistieron la fotometría de llama y la potenciometría y posteriormente solo la potenciometría. Los indicadores de prestación analítica han mejorado paulatinamente hasta obtener un CV del 2% y un sesgo del 2% en el año 2018.
ALT (Figura 2C): al igual que en otros enzimas, la desaparición de métodos colorimétricos produjo una reducción de la imprecisión inter-laboratorios, desde CV cercanos al 50% hasta CV inferiores al 5%. También el sesgo disminuyó espectacularmente.
TSH (Figura 2D): en 1999 se inició el programa de hormonas, utilizándose entonces diferentes métodos analíticos con un CV del 12% y un sesgo del 30%. A partir del 2010, con el uso mayoritario de métodos luminiscentes, el CV ha disminuido al 5% y el sesgo al 15%.

### Comparación de los programas EQA de la SEQC^ML^ con otros programas EQA

En la Tabla 1 se muestra la imprecisión inter-laboratorios obtenida en el año 2018 por los programas SEQC^ML^ (control no conmutable) [8], el programa holandés *Stichting Kwaliteitsbewaking Medische Laboratoriumdiagnostiek* (SKML) (control conmutable) [9], el belga *Empower project* (control conmutable) [10] y el alemán *Referenzinstitutfür Bioanalytik* (control no conmutable) [11], para algunas magnitudes biológicas de bioquímica en suero. En general los CV son similares entre los distintos programas, excepto para ALT (CV entre 4% y 10%) y LDH (CV entre 2.6 y 9.0%).

**Tabla 1:** Imprecisión inter-laboratorios (CV%) obtenida por varios programas europeos en el año 2018.

Magnitud	SEQC (NC) [8]	SKML (C) [9]	*Empower project* (C) [10]	*RfB* (NC) [11]
Albúmina	4,5	4,0	–	4,7
ALT	4,9	10,0	9,1	5,5
AST	5,0	7,5	3,3	5,7
Calcio	2,3	2,0	–	2,7
Creatinina	5,7	5,5	–	4,4
Fosfato	3,3	4,0	–	3,6
GGT	5,7	8,5	6,9	4,8
Glucosa	3,4	4,0	–	3,4
LDH	5,1	9,0	2,6	4,4
Magnesio	3,4	6,0	–	4,0
Potasio	2,0	1,0	1,2	2,3
Sodio	1,6	0,9	0,8	1,9
Urato	2,6	3,0	–	4,0
Urea	3,5	4,0	–	4,8

C, muestras conmutables; NC, muestras no conmutables.

En la Tabla 2 se compara visualmente la DP% respecto al grupo par obtenido en los programas SEQC^ML^ y en el ProBioQual (Francia) [12]; ambos utilizan material no conmutable y como valor diana el grupo par. Para este indicador se observan mejores resultados en el programa de la SEQC^ML^ (DP% visualmente inferiores).

**Tabla 2:** Error total analítico (DP% respecto al grupo par) obtenido por el percentil 90 de los participantes en dos programas europeos.

Magnitud	SEQC^ML^ [8] 2018	PBQ [12] 2015
Fosfatasa alcalina	10,4	15,1
ALT	7,5	11,7
Amilasa	7,2	9,1
AST	7,8	10,8
Bilirrubina	8,4	12,0
Calcio	4,3	5,7
Colesterol	5,5	6,6
Creatina quinasa	8,3	11,8
Cloro	3,9	5,6
Creatinina	9,2	11,0
GGT	9,7	14,1
Glucosa	5,0	7,3
Potasio	3,2	4,6
LDH	9,9	15,2
Lipasa	11,0	13,3
Magnesio	6,0	10,1
Sodio	2,7	3,6
Fosfato	5,9	8,2
Triglicérido	6,5	8,6
Urato	5,4	5,9
Urea	6,4	12,5

Controles no conmutables.

Valor diana: media del grupo homogéneo.

En la Tabla 3 se presentan los resultados del año 2018 del sesgo (DP%) obtenido en tres programas que usan controles conmutables con valores asignados por métodos de referencia certificados (SEQC^ML^-SCR, SKML y *Empower project*) [8, 10, 13]. Los valores que se muestran corresponden a la desviación porcentual entre la media del grupo par más frecuente y el valor de referencia. Los datos corresponden a enzimas determinados por métodos recomendados por IFCC. Se observa un menor sesgo (DP% visualmente inferior) en los resultados de los programas SEQC^ML^ y SKML.

**Tabla 3:** Sesgo (DP%) respecto al método de referencia obtenido para enzimas determinados por los métodos recomendados por IFCC (2018).

Magnitud	SEQC [8]	SKML [10]	*Empower project* [13]
Fosfatasa alcalina	−6,0	−10,0	−15,0
ALT	−16,0	−14,0	−25,0
AST	−12,0	−11,0	−26,0
Creatina quinasa	3,0	4,0	–
GGT	−5,5	−8,0	−15,0
LDH	−8,0	−5,0	−18,0

En la Tabla 4 se muestran las observaciones publicadas en diferentes estudios [8, 13–16] respecto a la comparabilidad de resultados entre sistemas método-instrumento, utilizando materiales control conmutables. Los autores consideran que existe una buena armonización para urea, colesterol, glucosa, urato y potasio y una pobre estandarización para creatinina, calcio, magnesio, cloro y sodio; mientras que los resultados en proteína y fosfato son discrepantes. Sin embargo, es importante recalcar que estas organizaciones utilizaron distintas EPA (estado del arte, VB), para considerar el grado de armonización de estas magnitudes biológicas.

**Tabla 4:** Grado de comparabilidad entre sistemas método-instrumento según varios estudios.

Magnitud	SCR SEQC [8]	SKML [13]	Empower [14]	Miller [15]	Van Houcke [16]
Urea	–	SI	–	SI	–
Colesterol	--	SI	SI	SI	–
Glucosa	DEP	SI	SI	SI	–
Creatinina	NO	NO	DEP	–	–
Fosfato	–	–	SI	DEP	–
Proteína	DEP	DEP	SI	–	SI
Urato	SI	SI	SI	SI	–
Calcio	DEP	NO	NO	–	DEP
Magnesio	DEP	DEP	DEP	DEP	DEP
Cloro	DEP	NO	DEP	NO	–
Potasio	SI	SI	SI	SI	–
Sodio	NO	NO	SI	NO	–

SI, sistemas método-instrumento estandarizados; NO, sistemas método-instrumento no estandarizados; DEP, algunos sistemas están estandarizados y otros no.

## Discusión

El material control de un EQA ha de ser similar a las muestras de los pacientes y el valor diana debería determinarse por métodos de referencia certificados [3]. Solo uno de los 29 programas de la SEQC cumple actualmente estas características (SCR), siendo las principales dificultades la no existencia de materiales control conmutables ni de métodos de referencia para todas las magnitudes biológicas del laboratorio clínico.

Si el programa EQA utiliza material conmutable con valor asignado por métodos de referencia, puede evaluar la verdadera exactitud del laboratorio individual, evaluar métodos analíticos, así como estimar el sesgo y promover la estandarización. En caso contrario, solo puede evaluar al laboratorio individual y comparar grupos homogéneos.

Los indicadores de la prestación analítica: ET analítico, imprecisión y sesgo calculados, asi como el tipo de las EPA utilizadas en los programas de la SEQC^ML^ para evaluar a los participantes siguen la recomendación de la 1^a^ Conferencia Estratégica EFLM (Milán 2014) [17].

Los laboratorios participantes han mejorado su prestación a lo largo de los años, como indican el mayor grado de cumplimiento de las EPA (Figura 1) y las desviaciones decrecientes respecto al valor diana obtenidas. Probablemente el haber utilizado el criterio derivado de la VB para evaluar los resultados favoreció esta mejora.

A pesar de las críticas sobre la utilización de la VB como especificación de la prestación por ser demasiado estricta [17], en este trabajo se evidencia que son alcanzables por los laboratorios, para muchas magnitudes biológicas.

La disminución de la dispersión de métodos, el abandono de métodos obsoletos (enzimas) y el uso de la quimioluminiscencia en inmunoensayos (Figura 2) son factores que favorecen la mejora de las prestaciones.

La comparación de los programas EQA de la SEQC^ML^ con otros programas de distintas organizaciones es difícil porque los informes solo son accesibles a los participantes; por ello, en este estudio se presentan datos únicamente de programas que publican sus resultados en revistas internacionales o en páginas web de libre acceso. Otras dificultades radican en las diferencias en los materiales control distribuidos, en el criterio para formar grupos homogéneos, en los cálculos realizados y en las especificaciones de la calidad aplicadas para evaluar los resultados.

Así las DP% inferiores de SEQC^ML^ comparadas con ProBioQual (Tabla 2) probablemente se deban a que el programa español utiliza como especificación la VB, mientras que el programa francés utiliza la prestación actual de los métodos, que es más permisiva.

En cambio, las discrepancias observadas en los tres programas que usan controles conmutables con valores asignados por métodos de referencia certificados (Tabla 3) no se pueden explicar a la luz de los datos disponibles en este estudio.

Cuando las EPA están basadas en el estado del arte, se ha podido constatar que la mayoría de los programas no definen el “estado del arte” como la *mejor prestación* posible de los métodos usados por los participantes, tal como se recomendó en la 1^a^ Conferencia estratégica EFLM de Milán [18], sino como la *prestación actual* de los métodos. Esta es una dificultad más para alcanzar la armonización.

Los datos mostrados en este estudio ponen de manifiesto que, a pesar de la que las prestaciones mejoren continuamente dentro de cada programa y se estén realizando esfuerzos para la armonización, los resultados obtenidos en diferentes programas para algunas magnitudes biológicas, todavía no están armonizados.

La armonización se conseguiría con el esfuerzo de todas las partes implicadas en la actividad del laboratorio médico: organizaciones internacionales que promueven normas y directrices, sociedades científicas que divulgan sus datos, la industria de diagnóstico *in vitro* que pone en el mercado métodos estandarizados y sistemas analíticos calibrados con patrones de trazabilidad documentada, y los laboratorios asistenciales que utilizan los mejores métodos con rigurosidad y protocolización.

Desde el año 1998 la comunidad internacional esta realizando esfuerzos para alcanzar la armonización, como queda plasmado en la siguiente documentación:

Directiva europea para la industria de diagnóstico *in vitro* (1998) que exige a los proveedores que aseguren la trazabilidad al nivel de referencia mas elevado, cuando sea posible
ISO 17511 (2003): *In vitro diagnostic medical devices-Measurement of quantities biological samples – Metrological traceability of values assigned to calibration and control materials.* Se publicó siguiendo los mismos requisitos de la directiva europea [19]
JCTLM (2002): creado por varias entidades internacionales que mantiene una base de datos de materiales de referencia, procedimientos analíticos y laboratorios de referencia de acuerdo con la norma ISO 17511 [20]
ICHCLR (2013): este consorcio tiene un portal global en el que se recoge la información actualizada sobre el estado de estandarización /armonización de las magnitudes biológicas y desarrolla protocolos para establecer la armonización de aquellas magnitudes biológicas para los que no existen materiales y métodos de referencia [21]
Nueva norma ISO/NP 21151 en desarrollo, en la que se defina los protocolos a seguir para asegurar la armonización de las magnitudes sin materiales ni métodos de referencia [19]

Los programas EQA juegan un importante papel en la vigilancia de la armonización de las prestaciones del laboratorio médico, pero se debería potenciar el uso de controles conmutables con valores de referencia certificados siempre que fuera posible y aplicar las especificaciones de la prestación analítica recomendadas.

## Conclusiones

Los laboratorios participantes en los programas EQA de la SEQC^ML^ han mejorado notablemente su prestación a lo largo de los 30 años de experiencia.

Las especificaciones derivadas de la variación biológica son alcanzables por muchos laboratorios en nuestro país. Para las magnitudes biológicas sin datos de VB conocidos, se utiliza el estado del arte actual y no el mejor posible.

Los organizadores de programas EQA también deberían sumarse al esfuerzo de armonización, facilitando la información sobre sus resultados a otros profesionales interesados y facilitando la comparación entre las organizaciones.

## Supplementary Material

Supplementary Material Details

## Material Suplementario

La versión en línea de este artículo ofrece material suplementario (https://doi.org/10.1515/almed-2019-0024).
