# Supplementary material for: Programas de garantía externa de la calidad SEQCML. Evolución de las prestaciones analíticas de los laboratorios clínicos a lo largo de 30 años y comparación con otros programas
Source: Adv Lab Med. 2020 May 4;1(2):20190024. [Article in Spanish] doi: 10.1515/almed-2019-0024 (PMC10159287; doi:10.1515/almed-2019-0024)
Supplement: Supplementary file 1 — Supplementary Material Details [file j_almed-2019-0024_suppl.docx]

Supplemental Figura 1. Informe para el laboratorio individual de los programas de categoría 4. Años 2003 y 2019


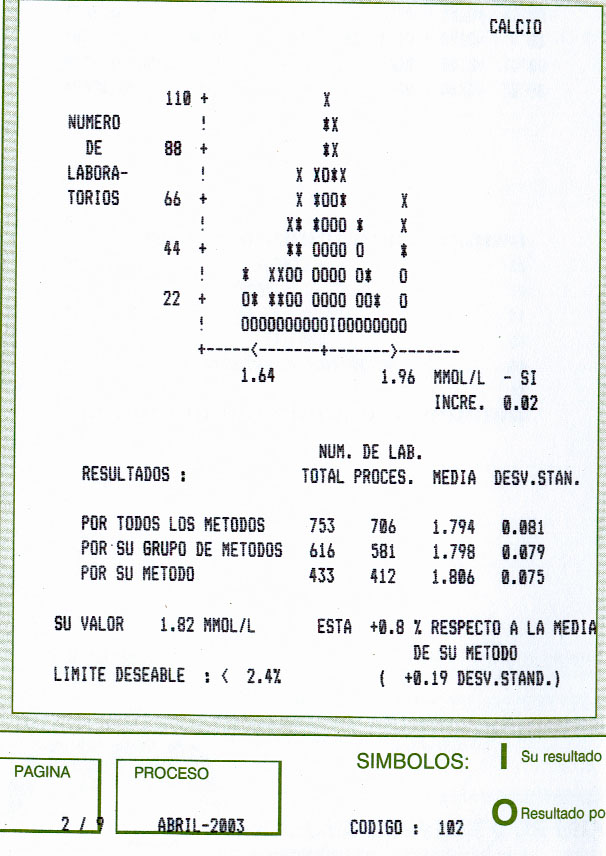

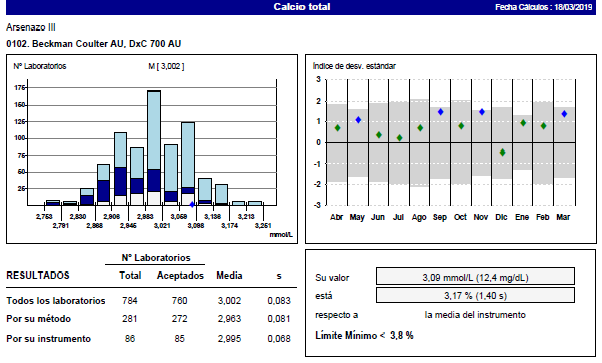


Supplemental Figura 2. Informe para el laboratorio individual de los programas de categoría 1


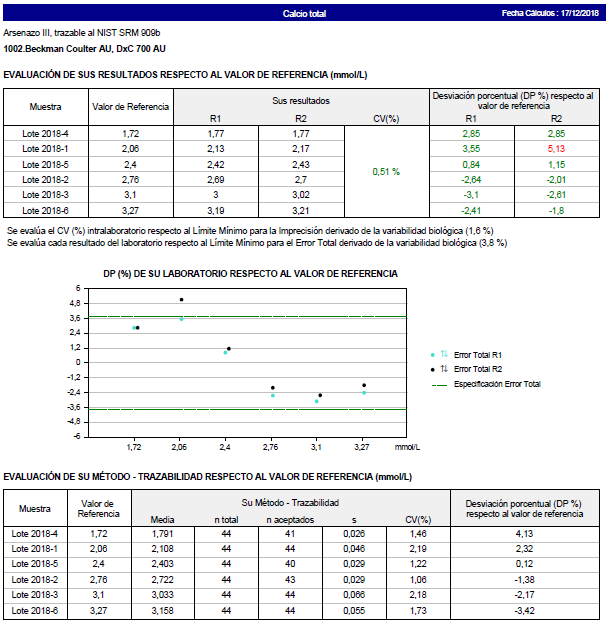


Supplemental Figura 3. Evolución del percentil 90 de las DP% respecto al grupo homogeneo (ET analítico) en el programa de hormonas
